# Concurrent Nivolumab‐Induced Myocarditis and Myasthenia Gravis: A Case Report

**DOI:** 10.1155/crom/9486566

**Published:** 2026-03-10

**Authors:** Rajat Gupta, Noorine Plumber, Jae Lee, Barath Prashanth Sivasubramanian, Mohammad Eshaq Kyhan, Hardeep Singh, Sonu Gupta

**Affiliations:** ^1^ Department of Internal Medicine–GME, Northeast Georgia Medical Center, Gainesville, Georgia, USA; ^2^ Department of Medicine, Philadelphia College of Osteopathic Medicine, Suwanee, Georgia, USA, pcom.edu; ^3^ Department of Research and Quality Improvement, Graduate Medical Education, Northeast Georgia Medical Center, Gainesville, Georgia, USA

**Keywords:** heart failure, melanoma, myasthenia gravis, myocarditis, nivolumab

## Abstract

**Background:**

Immune checkpoint inhibitors (ICIs) such as nivolumab have improved 10‐year overall survival rates up to 43% in advanced melanoma. They carry a risk of severe immune‐related adverse events (irAEs) up to 9%–33%, including cardiotoxicity and neuromuscular complications. Acral melanoma is a rare subtype that is often diagnosed late and requires aggressive therapy with adjunctive immunotherapy. Here, we report a rare case of an elderly male who developed myocarditis and myasthenia gravis 3 weeks after receiving the first dose of nivolumab for stage IIB acral melanoma.

**Case Presentation:**

A 72‐year‐old male with a late diagnosis of Stage IIB acral melanoma of the left great toe underwent toe amputation and received adjuvant therapy with nivolumab as per NCCN guidelines. Within 21 days after the first infusion, he developed chest pressure, fatigue, and diplopia. Workup revealed new‐onset heart failure with an EF of 45%–50% with Grade 2 diastolic dysfunction, elevated troponin and NT pro‐BNP, and cardiac MRI findings consistent with severe Grade 3, immune‐related myocarditis. This presentation was complicated by a Mobitz Type 2 AV block, 10‐s asystole, and therefore required permanent pacemaker placement. The symptoms of fatigue and diplopia led to a diagnostic workup with electromyography, confirming Grade 3 nivolumab‐induced myasthenia gravis. He was treated with dexamethasone followed by a tapering dose of prednisone, and one cycle of IVIG at 0.4 g/kg for 5 days, leading to symptom resolution and improvement of EF to 60%–65%. However, 2 months later, he developed atrial fibrillation with a rapid ventricular rate, with device check showing 100% burden, therefore requiring hospitalization and subsequent cardioversion.

**Conclusion:**

Nivolumab‐induced myocarditis and myasthenia gravis require high clinical suspicion to make an early diagnosis and ICI discontinuation and aggressive immunosuppressive treatment. Due to early onset and rapid progression to conduction abnormalities and the development of new arrhythmogenic foci, immunotherapy‐related myocarditis requires close monitoring. This case also highlights the importance of multidisciplinary management and individualized risk‐benefit assessment when considering rechallenge with ICIs.

## 1. Background

The incidence of primary cutaneous melanoma has gone up by 320% in the United States since 1975 and is still the most fatal form of cutaneous tumors [1]. Acral melanomas are usually found in nail beds and constitute only 1%–3% of all melanoma cases; therefore, the diagnosis is often delayed. [28] They are often treated with surgical resection, radiation, and immunotherapies. Immune checkpoint inhibitors (ICIs) are monoclonal antibodies that work by blocking checkpoint proteins on immune and cancer cells, therefore aiding T cells in killing the cancer cells. ICIs like nivolumab have proven to improve the overall survival, with 10‐year overall survival rates being 43% with nivolumab plus ipilimumab and 37% with nivolumab monotherapy [2]. Nivolumab is a potent immunotherapy that targets Programmed Cell Death Protein 1 (PD‐1) immune receptors and thus obstructs the signal that impedes the activation of T cells against cancerous cells [3]. Because of the drug′s T cell stimulating mechanism of action, side effects from nivolumab therapy are frequently autoimmune and may impact any organ system [4]. Adverse events related to the use of ICI therapy are defined as immune‐related adverse events (irAEs) and are graded according to the Common Terminology Criteria for Adverse Events (CTCAE) Version 5.0 [5]. As per a retrospective analysis, 43.4% had an incidence of irAEs, with 92.1% of those being Grade 1–2 and only 7.9% being Grade 3 or higher. Multi‐organ irAEs are a recognized phenomenon with ICIs however most of the events are Grades 1–2 [6]. There is limited literature available on nivolumab‐induced dual irAEs, and especially the co‐occurrence of Grade 3 cardiotoxicity and neurotoxicity [7–11].

We present a rare case of Stage II B acral melanoma that developed nivolumab‐induced severe Grade 3 immune‐related myocarditis and Grade 3 myasthenia gravis.

## 2. Case Presentation

A 72‐year‐old male presented with a past medical history of metabolic dysfunction–associated steatohepatitis (MASH), hypertension, right temporal cavernoma, and esophageal dysphagia. He had a 2‐year history of a skin lesion on the left great toe, which was initially treated as a chronic fungal infection and later diagnosed as Stage II B acral melanoma 5 months ago. He underwent the left great toe amputation, given the melanoma, and was also found to have osteomyelitis of the toe. He was initially managed with daptomycin and ceftazidime and later transitioned to minocycline for chronic suppressive therapy. The patient received his first immunotherapy with nivolumab 3 weeks ago, as indicated for melanomas > 4 mm in size as per National Comprehensive Cancer Network (NCCN) guidelines [12].

**Figure 1:**
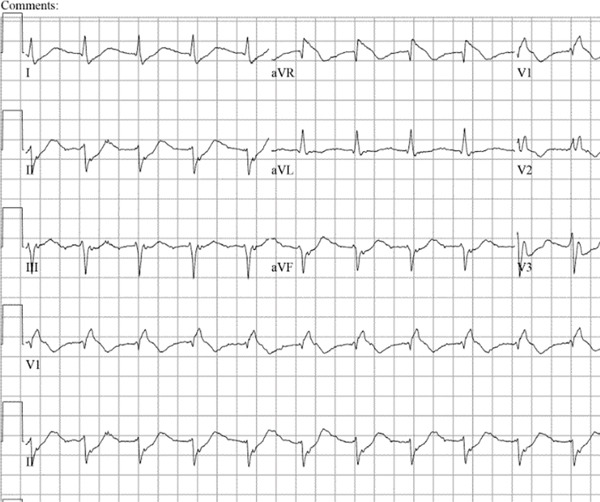
EKG showing diffuse ST elevation suggestive of myocarditis.

Three weeks after the infusion, the patient presented to the emergency department with complaints of a 2‐week history of intermittent, nonexertional, midsternal chest pressure, polyarthralgia, fatigue, along with new‐onset diplopia. He was hemodynamically stable, but EKG was suggestive of sinus rhythm, ST elevation in aVR, and ST depression in Leads I, II, aVF, V3, V4, V5, and V6 with right bundle branch block (Figure 1). Neurological examination indicated diplopia on looking towards the left and variable ptosis, but no other neurofocal deficit was noted. His TTE was suggestive of an EF of 46%–50% with Grade 2 diastolic dysfunction, suggestive of new‐onset heart failure with mild reduced ejection fraction. Patient′s Troponin I peaked at 12.012 ng/mL, and he was taken up for left heart catheterization, which showed nonobstructive disease in PDA, LAD, and circumflex coronary arteries (30% mid‐LAD, 40% midcircumflex, and 50% mid‐PDA). Cardiac MRI findings were compatible with an LVEF of 53%, a mixed picture of a small ischemic infarction of the basal inferolateral segment and acute myocarditis (in the setting of high T2 nonischemic LGE pattern), along with suspected myocardial edema (Figure 2a,b). ESR and CRP were within normal limits, but CPK was elevated. Therefore, the patient met the diagnostic criteria for severe Grade 3 immune‐related myocarditis. Minocycline was discontinued, and he was started on 1 g of Solumedrol with improvement in his symptoms. However, on the 5th day of admission, he became lightheaded, and the telemetry was suggestive of bradycardia with HR in the 40–50s. EKG showed bradycardia with Mobitz Type 2 and junctional rhythm. He had a 10‐s asystole event with spontaneous ROSC. The cath lab was activated for transvenous pacing. A temporary pacemaker was placed, and a dual‐chamber biventricular pacemaker was placed a day later.

Figure 2(a, b). Cardiac MRI showing high T2 nonischemic late gadolinium enhancement pattern.

**Figure:**
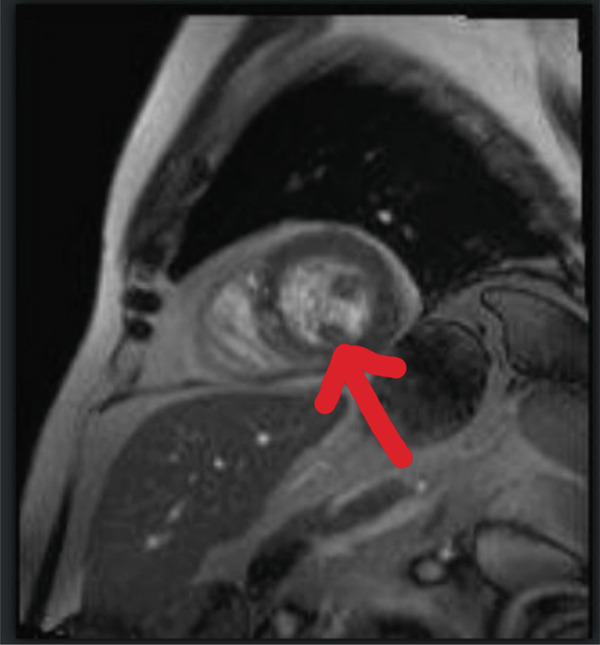
(a)

**Figure:**
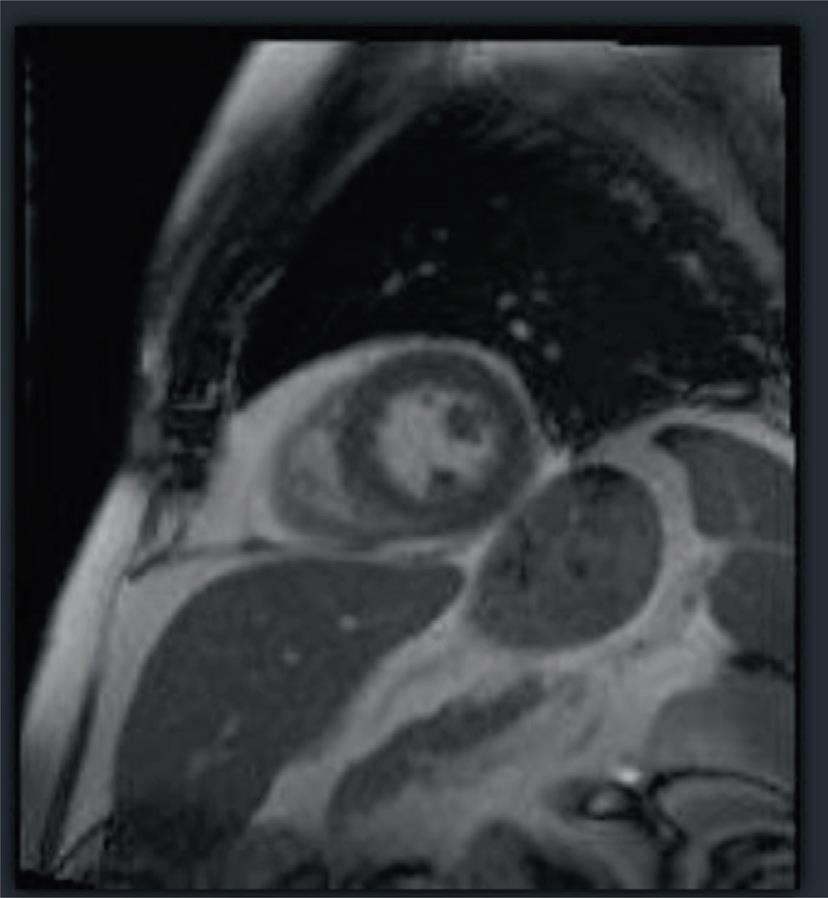
(b)

His workup for myasthenia gravis was suggestive of negative AchR antibodies, with MRI and MRA of the brain grossly unremarkable except for a stable, 11‐mm cavernous malformation in the right inferior frontal gyrus. The single fiber electromyography was consistent with myasthenia gravis. However, advanced serological testing including anti‐MuSK or LRP‐4 antibodies was not tested. The patient′s symptoms correlated with Grade 3 irAE and thus was initiated on high‐dose corticosteroids, IVIG for 5 days, and was discharged home with pyridostigmine 60 mg, tapering doses of high‐dose steroids, along with Bactrim for *Pneumocystis jiroveci* pneumonia (PJP) prophylaxis.

Repeat TTE, 3 weeks from initial, suggested an EF of 50%–55% with normal LV diastolic function, hence indicative of recovered EF. The device check on the 8th day postimplant showed 99.9% biventricular paced rhythm and PVC burden of 2.7/h. But 4 weeks postplacement, the patient was found to have three episodes of VT, the longest of which lasted 34 s. At the 5th week, he was noted to have a new onset of atrial flutter with a 3.3% burden and a 15.9% burden of atrial fibrillation with RVR at the 6th week and a PVC burden of 27.6 per hour. He presented to the emergency department on the same day for acute heart failure exacerbation with worsening dyspnea, bilateral lower extremity pitting edema, and weight gain of 25 pounds. TTE showed an EF of 60%–65% with inaccessible diastolic function due to arrhythmia. Device check in the hospital showed 100% burden of atrial flutter with RVR. He was thus diuresed and taken up for TEE and direct cardioversion with successful restoration of sinus rhythm. He was net 11 L negative on discharge and sent home on metoprolol 25 mg twice daily, apixaban 5 mg twice daily, and furosemide 40 mg daily. Table 1 provides a timeline of all the above events.

**Table 1:** Patient timeline.

Time (relative)	Event	Key findings	Management/interventions
~2 years prior	Left great toe skin lesion	Initially treated as chronic fungal infection	—
~5 months prior	Diagnosis: Stage IIB acral melanoma	Lesion on left great toe	Left great toe amputation; intraoperative/clinical dx of osteomyelitis
Postoperative period	Osteomyelitis treatment	—	Daptomycin + ceftazidime, later transitioned to minocycline for chronic suppression
3 weeks prior to ED	First nivolumab infusion	Indication: melanoma > 4 mm (per NCCN)	Immunotherapy initiated
ED presentation (3 weeks after nivolumab; 2‐week symptom history)	Chest pressure, polyarthralgia, fatigue, new diplopia	Hemodynamically stable; EKG: sinus rhythm, ST↑ aVR, ST↓ I, II, aVF, V3–V6, RBBB; neuro: diplopia on left gaze; TTE: EF 46%–50%, Grade 2 diastolic dysfunction (new HFmrEF); Troponin I peak: 12.012 ng/mL	Taken for left heart catheterization → nonobstructive CAD (30% mid‐LAD, 40% midcircumflex, 50% mid‐PDA)
Hospital Day 0–1	Advanced cardiac imaging	Cardiac MRI: LVEF 53%; small ischemic infarct (basal inferolateral) + acute myocarditis (nonischemic LGE, high T2), suspected myocardial edema; ESR/CRP normal, CPK elevated	Dx: Severe (Grade 3) immune‐related myocarditis (ir‐myocarditis); minocycline discontinued; Solumedrol 1 g started
Hospital Day 5	Bradyarrhythmia and asystole	Telemetry: HR 40–50; EKG: bradycardia, Mobitz II, junctional rhythm; 10‐s asystole with spontaneous ROSC	Cath lab activated; temporary transvenous pacemaker placed
Hospital Day 6	Permanent device implantation	—	Dual‐chamber biventricular pacemaker implanted
Inpatient workup	Suspected myasthenia gravis (irAE)	AchR Ab negative; MRI/MRA brain: grossly unremarkable except stable 11‐mm cavernoma (right inferior frontal gyrus); EMG consistent with MG	High‐dose corticosteroids, IVIG x5 days
Discharge (postinitial admission)	Discharge meds	—	Pyridostigmine: 60 mg, tapering high‐dose steroids, Bactrim for PJP prophylaxis
3 weeks after initial TTE	Repeat TTE	EF: 50%–55%, normal LV diastolic function	—
Postimplant Day 8	Device check	99.9% biventricular paced; PVC burden 2.7/h	—
Postimplant Week 4	Ventricular tachycardia episodes	Three VT episodes, longest: 34 s	—
Postimplant Week 5	New atrial flutter	AFL burden: 3.3%	—
Postimplant Week 6	Atrial fibrillation with RVR	AF burden: 15.9%; PVC burden: 27.6/h	—
Same day (Week 6)	Acute heart failure exacerbation → ED	Dyspnea, BLE pitting edema, +25‐lb weight; TTE EF: 60%–65% (diastolic function inaccessible due to arrhythmia); device check: 100% AFL burden with RVR	Diuresis; TEE + direct cardioversion → successful sinus rhythm

## 3. Discussion

Nivolumab‐associated myasthenia gravis and its co‐occurrence with myocarditis were documented in limited case reports [7–11]. In a retrospective study that analyzed 67 cases of nivolumab‐induced MG, 31.3% of patients had concurrent myositis, and 14.9% had myocarditis. The mortality rate was 16.4%, due to myasthenia gravis complications, underscoring the severity of this adverse event [13]. Many studies have reported cardiac complications of myocarditis, complete heart block, and heart failure [14, 15]. The reported incidence of ICI‐induced myocarditis is low, with safety databases citing rates below 1% for nivolumab monotherapy (0.06%) and slightly higher for combination therapies (0.27%) [14, 15]. In this case of severe Grade 3 irAE of myocarditis and concomitant Grade 3 myasthenia gravis, it manifested 3 weeks after the first nivolumab infusion. This aligns with the reported median time from the start of ICI to development of symptoms of 23 days (IQR of 14–36 days) for nivolumab therapy alone [1]. Studies found that patients of younger age were at an increased risk of severe irAEs, whereas increased mortality and prolonged hospitalization (*p* < 0.001) were common among older patients [16, 17]. Younger patients had higher rates of colitis (16%) and hepatitis (11%), whereas older patients had higher rates of pneumonitis (18%) and myocarditis (18%) [16, 17].

The development of acute myocarditis was confirmed with cardiac MRI, followed by rapid progression to heart block and asystole, highlighting the severity of ICI‐related cardiotoxicity requiring urgent intervention [16, 18]. Management with high‐dose corticosteroids (methylprednisolone 1 mg/kg/day, with taper over 4–6 weeks) and intravenous immunoglobulin (one cycle of IVIG 0.4 g/kg/day for 5 days) led to significant symptom resolution and recovery of ejection fraction (EF 50%–55% at 3 weeks, 60%–65% at 6 weeks). This aligns with current management guidelines for Grade 3 irAEs, which recommend aggressive immunosuppression and IVIG for Grade 3 myocarditis and myasthenia gravis [5, 16]. However, the patient′s subsequent development of atrial fibrillation with rapid ventricular response and acute decompensated heart failure depicts the long‐term cardiac sequelae of ICI‐induced Grade 3 myocarditis. Rechallenging patients with ICI therapy in patients after resolution of toxicity necessitates consideration of several factors, including but not limited to, previous tumor response, duration of treatment, type and severity of toxicity, and time to toxicity resolution [16]. For patients who experienced rapid resolution of irAE after corticosteroid use, resuming ICI must be approached with shared decision‐making, whereas prolonged recovery may suggest a higher risk of irAE recurrence [16, 19].

Diagnosis of irAE MG in seronegative cases usually relies on a combination of clinical, electrophysiological, and/or advanced serological testing. For patients with Grade 3–4 myasthenia gravis irAEs, permanent discontinuation of ICIs is advised. For Grade 3 myocarditis irAE, the suitability of rechallenging with ICIs is still uncertain [16]. The co‐occurrence of myocarditis and myasthenia gravis underscores the importance of a multidisciplinary approach involving cardiology, neurology, and oncology.

## 4. Conclusion

In conclusion, nivolumab‐induced myocarditis and myasthenia gravis represent rare but life‐threatening complications of ICI therapy. Clinicians must maintain a low threshold for suspecting irAEs in patients presenting with cardiac or neurological symptoms during immunotherapy. Prompt diagnosis, cessation of ICIs, and aggressive immunosuppression are critical to improving outcomes. This case highlights the delicate balance between the therapeutic benefits of ICIs and their potential for severe toxicity, advocating for personalized monitoring strategies in melanoma treatment.

## Funding

No funding was received for this manuscript.

## Ethics Statement

The authors have nothing to report.

## Consent

Informed consent was obtained for the publication of deidentified information and case details.

## Conflicts of Interest

The authors declare no conflicts of interest.

## Data Availability

The data that supports the findings of this study are available from the corresponding author upon reasonable request.
